# Glutathione and Glutaredoxin in Redox Regulation and Cell Signaling of the Lens

**DOI:** 10.3390/antiox11101973

**Published:** 2022-10-01

**Authors:** Marjorie F. Lou

**Affiliations:** 1 School of Veterinary Medicine and Biomedical Sciences, Redox Biology Center, University of Nebraska-Lincoln, Lincoln, NE 68583, USA; mlou1@unl.edu; 2 Department of Ophthalmology, University of Nebraska Medical Center, Omaha, NE 68198, USA; 3 Department of Pharmaceutical Sciences, System College of Pharmacy, University of North Texas Health Science Center, Fort Worth, TX 76107, USA

**Keywords:** the ocular lens, cataract, reactive oxygen species (ROS), glutathione (GSH), protein-thiol mixed disulfide, redox regulation, thioltransferase (glutaredoxin), redox signaling, cell proliferation

## Abstract

The ocular lens has a very high content of the antioxidant glutathione (GSH) and the enzymes that can recycle its oxidized form, glutathione disulfide (GSSG), for further use. It can be synthesized in the lens and, in part, transported from the neighboring anterior aqueous humor and posterior vitreous body. GSH is known to protect the thiols of the structural lens crystallin proteins from oxidation by reactive oxygen species (ROS) so the lens can maintain its transparency for proper visual function. Age-related lens opacity or senile cataract is the major visual impairment in the general population, and its cause is closely associated with aging and a constant exposure to environmental oxidative stress, such as ultraviolet light and the metabolic end product, H_2_O_2_. The mechanism for senile cataractogenesis has been hypothesized as the results of oxidation-induced protein-thiol mixed disulfide formation, such as protein-S-S-glutathione and protein-S-S-cysteine mixed disulfides, which if not reduced in time, can change the protein conformation to allow cascading modifications of various kinds leading to protein–protein aggregation and insolubilization. The consequence of such changes in lens structural proteins is lens opacity. Besides GSH, the lens has several antioxidation defense enzymes that can repair oxidation damage. One of the specific redox regulating enzymes that has been recently identified is thioltransferase (glutaredoxin 1), which works in concert with GSH, to reduce the oxidative stress as well as to regulate thiol/disulfide redox balance by preventing protein-thiol mixed disulfide accumulation in the lens. This oxidation-resistant and inducible enzyme has multiple physiological functions. In addition to protecting structural proteins and metabolic enzymes, it is able to regulate the redox signaling of the cells during growth factor-stimulated cell proliferation and other cellular functions. This review article focuses on describing the redox regulating functions of GSH and the thioltransferase enzyme in the ocular lens.

## 1. Introduction: The Ocular Lens

### 1.1. The Structure and Function of a Vertebrate Ocular Lens 

**Figure 1:**
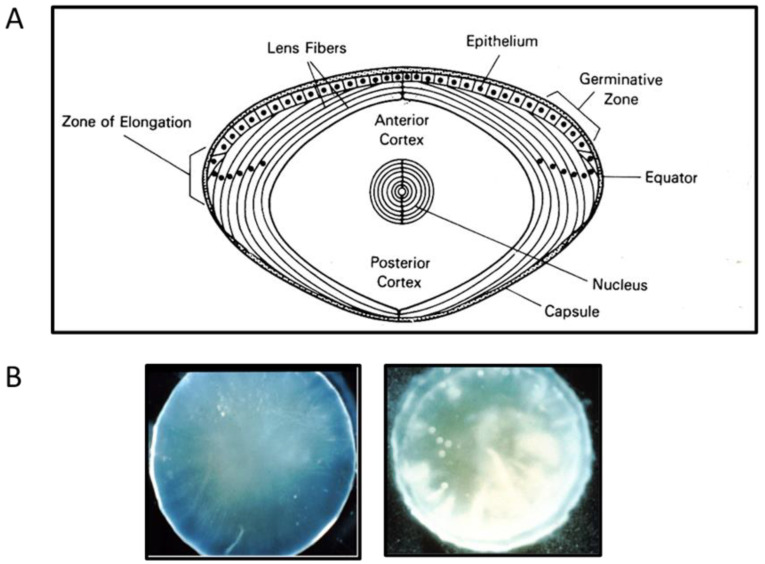
Schematic diagram and photos of a mammalian lens. (**A**) Diagram depicts the capsule, epithelium layer, outer cortical (younger), inner cortical (older) fiber cells, and nucleus regions of a mammalian lens. (**B**) Photos of a clear (left) and an opaque, cataractous (right) human lens.

The ocular lens is a unique body organ which functions in the focusing of light on the retina. It is avascular, has no nervous system and is enclosed in a flexible capsule. It grows throughout life with new cells generated from the single layer of epithelial cells located immediately inside the anterior capsule. Cells at the periphery of this layer continuously divide, elongate and differentiate into a ribbon-like fiber cells. These cells lose their nuclei and most of the organelles as they mature and are compacted and packed into the lens center over pre-existing cells. Thus, the fiber cells are concentrically arranged by age. The outer cells inside the epithelial layer are the youngest fiber cells and have a strong metabolic activity, followed by the inner less active fiber cell layer called the cortex, and the compact center area of oldest and inactive cells called the nucleus (Figure 1A). Nearly 40% of human lens wet weight, and almost all the dry weight are made of lens specific crystallin protein family, including α-, β- and γ-crystallins. These proteins are maintained in a reduced state and are packed in a specific short-range order to provide a smooth gradient of refractive index, increasing from the periphery to the center of the lens [1]. 

### 1.2. Pathology of an Eye Lens: Cataract 

The major pathological change of a lens is the loss of its transparency that affects visual function of the eye. This condition is called cataract, or opacity of the crystallin lens (Figure 1B). The causes of lens opacification are manyfold including congenital defects, diabetes, and most of all, aging. Several review articles describing the nature and the mechanism of the age-related (senile) cataract have been published, and almost all indicate that oxidative damage, resulting in protein aggregation, is the main culprit [2,3,4,5,6,7,8,9]. The mechanism of aging-related cataract is the main subject to be focused on in this review.

## 2. Sources of Oxidative Stress and the Damage to the Lens

The main sources of oxidative damage to the lens are the reactive oxygen species (ROS), which are known to be toxic and harmful by-product of living in an aerobic environment. These species include superoxide anion (O_2_^−^), hydroxyl radical (OH^−^) and hydrogen peroxide (H_2_O_2_). The first two are very unstable and short lived while the last one is freely diffusible and relatively long-lived. ROS can be generated internally by several enzyme systems, such as cytochrome P450, NADPH oxidase, peroxisomes and lipooxygenases and also by the mitochondria. They can be produced exogenously from the environment, including ultraviolet (UV) light, ionizing radiation, chemotherapeutics, inflammatory cytokines or environmental toxins [3,4,5,8]. Of the exogenous sources, UV light and ionizing radiation are the most damaging because of prolonged exposure of the lens during the long lifespan of the individual [10].

## 3. The Built-in Reducing System against Oxidative Stress in the Lens: Glutathione (GSH)

Like other tissues/organs in the body, the lens is rich in antioxidants and detoxification enzymes for protection against oxidative damage. Glutathione (GSH) in particular is present in very high level at a range of 2–17 µmole/g, similar to red blood cells [11,12,13]. It is an important reducing molecule for detoxifying ROS, as well as a cofactor in several redox enzyme reactions, such as glutathione reductase, to maintain the lens proteins in the reduced and soluble state necessary for transparency, and to protect the lens membrane SH groups in cation transporters for proper permeability function. The lens has an efficient de novo biosynthetic system for GSH production in the epithelial and cortical layers through the classical pathway found in other tissues. 

The amino acid pools of glutamic acid, cysteine and glycine for GSH synthesis are provided through respective transport systems from the aqueous humor as well as extracellular GSH recycling from the γ-glutamyl cycle into the lens by the epithelium [13]. Lim and associates in recent years have identified glutamate and glycine transporters [14], and a cysteine/cystine transporter [15] in the lens. In addition, other studies have suggested that GSH may be directly transported into the lens via a GSH transporter [16], but the result was not conclusive. Years later, Li et al. [17] suggested the presence of a dynamic system in the apical side of the lens epithelium to regulate the GSH pool using in situ synthesis to modify the uptake of GSH from the aqueous. This direct GSH transport mode is particularly necessary when the lens GSH synthesis machinery is impaired, as in the aging human lens. Recent studies of Fan and associates [18] have provided strong evidence that vitreous, not aqueous, is likely the major GSH supply source for the lens cortical fiber cells. The influx appears to take place via a connexin hemichannel whose opening is gated. This new finding provides a logical explanation, in part, that vitrectomy, a necessary surgical procedure to remove vitreous from a diseased eye, often causes these eye patients to develop cataract.

**Figure 2:**
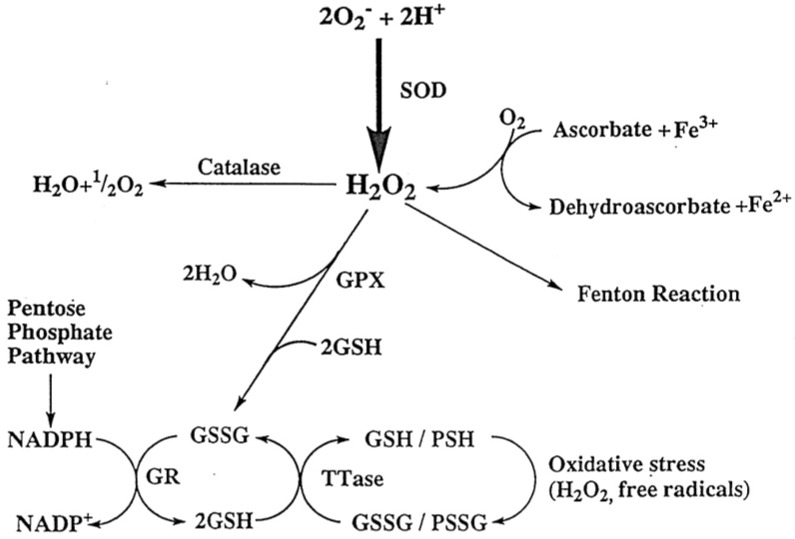
The reactive oxygen species and the antioxidant systems in the lens. H_2_O_2_-generated by the dismutation of superoxide anion or by the reaction between ascorbate and Fe^3+^ can be degraded by several pathways. These include Catalase, glutathione peroxidase and the Fenton reaction. The decrease in the SH/S-S ration by oxidation can be reversed by the glutathione reductase (GR)-pentose phosphate shunt cycle and by thioltransferase (TTase). These mechanisms protect the lens from oxidative damage. GR: glutathione reductase; GPx: glutathione peroxidase; SOD: superoxide dismuase; TTase: thioltransferase. Reprinted with permission from Lou, PRER (2003); Copyright Elsevier 2003 [8].

GSH is an effective antioxidant for removing oxidants in the lens especially the toxic H_2_O_2_. For instance, when the very abundant ascorbic acid in the aqueous humor is oxidized to dehydroascorbic acid, H_2_O_2_ is produced and diffuses into the neighboring lens. In such case, GSH in the lens can detoxify it, resulting in its oxidation to the GSSG disulfide. GSSG is then reduced back to GSH by the coupling reaction with glutathione reductase and NADPH. The hexose monophosphate shunt (pentose shunt) is the major hydrogen donor of NADPH (Figure 2). 

In addition to GSH, there are several important antioxidant enzymatic systems in the lens, including glutathione peroxidase, catalase, and superoxide dismutase [4]. However, these subjects are outside the scope of this current review. 

## 4. Hypothesis on the Mechanism of Senile Cataract Formation: The Role of Protein S-S-Glutathione Mixed Disulfides

**Figure 3:**
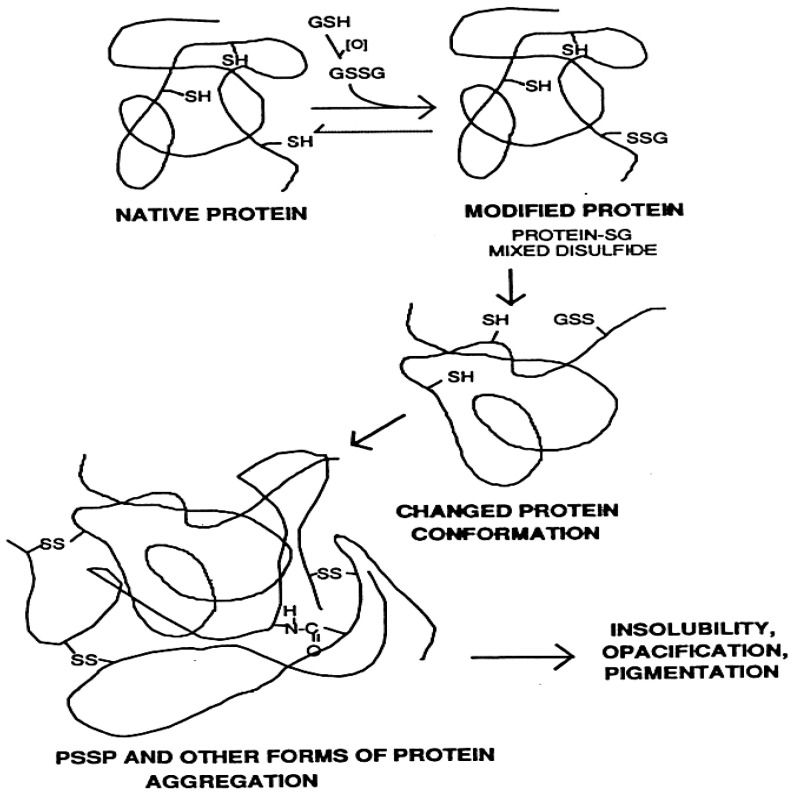
Hypothesized mechanism of the role of protein-S-S-glutathione in lens protein aggregation during cataract formation. Initial step, lens GSH is oxidized to GSSG and accumulated. 2nd step, GSSGs conjugate randomly with the thiol groups of crystallin proteins (native protein) to form protein-S-S-glutathione mixed disulfides (PSSGs). 3rd step, PSSG formation causes conformation change in crystallin proteins. Final step, Protein-S-S-protein (PSSP) crosslinking and/or other protein modifications occur with protein–protein aggregation. These changes lead to protein insolubility, opacification, and even pigmentation. Reprinted with permission from Lou, PRER (2003); Copyright Elsevier 2003 [8].

The major structural proteins in the lens are the crystallins, which are rich in thiol groups and need to be in the reduced state to maintain lens transparency. Under a constant or even elevated oxidative stress, a young lens with a rich GSH pool is able to completely detoxify the agents responsible. However, an older lens with a lower GSH level and a less efficient GSSG to GSH recycling system, can be overwhelmed by the stress, resulting in GSSG accumulation. In such a situation, GSSG may interact randomly with lens protein thiols to form protein-S-S-Glutathione mixed disulfides (PSSG), which if not dethiolated in time, the added extra charge and weight will change the protein conformation and allow protein–protein disulfide formation and other protein modifications leading to protein–protein aggregation. When the aggregates are large enough, they will scatter light and/or become insoluble leading to lens opacification (cataract). This possible scenario depicted in Figure 3 has been proposed by Lou et al. [19] and has been studied extensively in their laboratory [20,21,22,23,24,25,26,27]. 

Several key observations strongly support the hypothesis. First, PSSG formation appears to be oxidative-stress specific as it accumulates in several oxidant-induced, but not in hyperglycemic-induced cataract models [8,20]. Second, during aging, human lens GSH is gradually lost with concomitant elevation of PSSG. PSSP in the human lens is also gradually increased with increasing age [22,28]. Third, PSSG accumulation in human cataractous lenses is closely associated with the severity of the opacity and pigmentation, and inversely related to the level of GSH in the same tissue [21,27]. Fourth, in a pig lens organ culture model, mass spectrometry analysis of γB crystallin protein isolated from a H_2_O_2_-induced cataractous lens showed that PSSGs were formed with the buried SH groups after the initial PSSG modification of the exposed protein thiols. Such findings strongly suggest that random PSSG formation actually allows the opening of protein conformation for further modifications [26]. Fifth, in a H_2_O_2_-induced rat lens organ culture model, PSSG levels increased with the time of oxidant exposure (1–3 days) correlating with increased opacity; however, when H_2_O_2_ was removed after 24 h, the lens incubated in H_2_O_2_-free medium for additional days showed normal basal level of PSSG with resumed transparency of the lens, similar to the morphology and low PSSG level found in a control lens without H_2_O_2_ exposure [24,29]. The findings in these studies strongly support the hypothesis that PSSG is likely a major culprit for protein–protein aggregation and lens opacification (Figure 3). 

Although accumulation of PSSG in human cataractous lenses has been reported by several pioneer lens researchers [30,31,32,33], the significance was not appreciated until the technique for an accurate quantification of PSSG in a small sample size was developed by Lou et al. [20]. With this ion-exchange chromatographic analytical technique, besides PSSG, a second protein thiol modified by oxidized cysteine (cystine) forming protein-S-S-cysteine mixed disulfide (PSSC) [19,20,21,22,23,24,25,26,29], plus a minor component of protein thiols modified by γ-glutamylcystine forming protein-S-S-γ-glutamylcysteine (PSSGC) were also found in the human cataractous lenses [23,27], albeit much less than PSSG. The formation of PSSGC is likely associated with the malfunction of GSH biosynthesis in the pathological state of a lens. However, the presence of PSSC is intriguing as it is mostly concentrated in the inactive nuclear portion (center) of the lens and is closely associated with a later stage, more severe cataract formation [8]. Additional research in this area is needed to clarify the role of PSSC in cataractogenesis. The readers are encouraged to read other review articles on this general topic [8,9,34,35].

## 5. Redox Control in the Lens—Discovery of Thioltransferase (Glutaredoxin 1)

**Figure 4:**
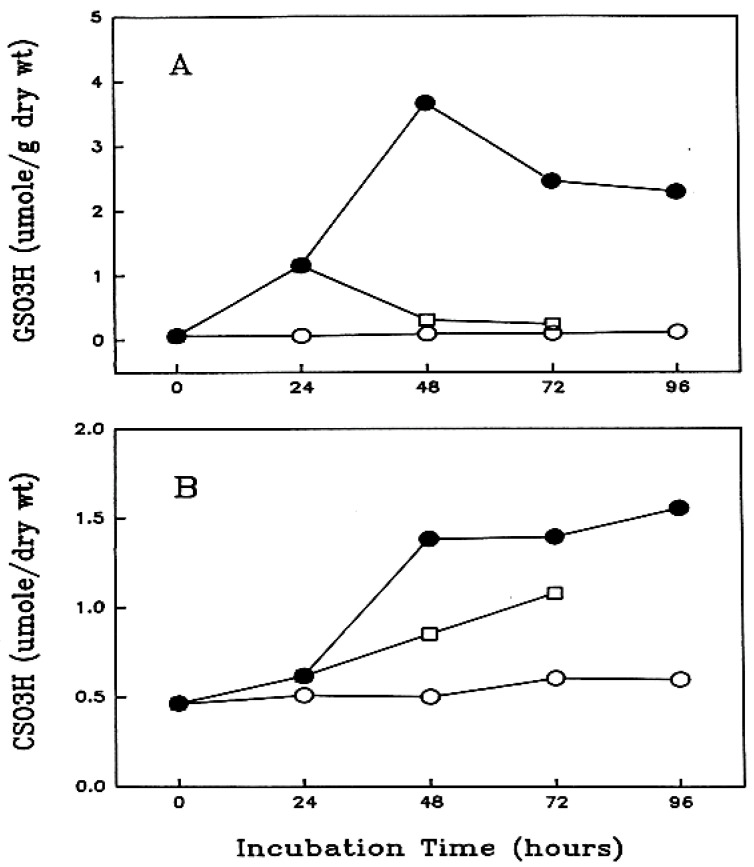
Formation and recovery of protein-thiol mixed disulfides in rat lenses during long term incubation with H_2_O_2_ (0.5 mM). The lenses were exposed to a constant concentration of H_2_O_2_ (0.5 mM). (**A**) Protein-S-S-glutathione (measured as GSO_3_H). (**B**). Protein-S-S-cysteine (measured as CSO_3_H). Five lenses of the same group were pooled and used for the analysis. Data are expressed as μmole/g dry wt., Mean ± SD, *n* = 5. The standard deviation values of the control and the recovery groups are too small to show in the plot. -○-: control group (untreated); -●-: H_2_O_2_ treated group (continuous H_2_O_2_ exposure); ⟥: recovery group (only first 24 h H_2_O_2_ exposure). Reprinted with permission from Cui and Lou, EER (1993); Copyright Elsevier 1993 [24].

As described above, a surprise finding [24] was that the elevated PSSG and concomitant transparency loss in a rat lens exposed to H_2_O_2_ stress could be completely reversed by removal of the H_2_O_2_ (Figure 4A) in the early stage of cataract formation (24 h). This reversibility was time- and dose-dependent of the oxidative stress condition. The reversal was specific for PSSG, not PSSC (Figure 4B). The same study also found that once PSSC and PSSP occurred (0.5 mM H_2_O_2_ for 48 h), the reversibility of PSSG was no longer feasible. 

This interesting discovery prompted us to search for a possible explanation, such as, is there a possibility that an endogenous enzyme system in the lens that can reduce PSSG to free protein-SH, allowing the lens to return to normalcy? The human lens is constantly at risk from oxidative stress and yet lens opacity mainly occurs late in life. Therefore, the human lens must have a repair system of sort to combat oxidative damage to lens protein/enzyme and to restore cellular functions. A likely candidate is the thiol-disulfide regulator defense system against oxidative stress called thioltransferase (or glutaredoxin E. C. 1.8.4.3) that is widely distributed from *E. coli* [36], to mammalian tissues such as the liver [37,38], placenta [39] and red blood cells [40,41]. 

Thioltransferase (TTase) belongs to the family of thiol-disulfide oxidoreductase, which also includes thioredoxin and protein disulfide isomerase [41]. TTase is a low molecular weight cytoplasmic enzyme that catalyzes reversible reduction in protein disulfides and thiol disulfide interchange between glutathione and protein sulfhydryls. The catalytic mechanism involves two steps as proposed by Allen and Mieyal [42]. First, TTase can dethiolate (deglutathionylate) the protein-S-S-G mixed disulfide (PSSG), releasing the reduced protein (PSH) and forming an intermediate of glutathionylated TTase, or TTase-glutathione mixed disulfie (TTase-S-S-G). The glutathionylated intermediate is then reduced by GSH, restoring TTase and producing oxidized glutathione (GSSG). GSSG is then reduced back to GSH via a coupling reaction with NADPH and glutathione reductase. 

Step 1: PSSG + TTase —> PSH + TTase-S-S-G;

Step 2: TTase-S-S-G + GSH —> GSSG + TTase.

In 1996, the Lou laboratory confirmed the presence of thioltransferase in the bovine lens [43], in which a partially purified enzyme showed similar catalytic function with heat and oxidative stress-resistant properties as the enzyme from the liver [38] or red blood cells [41]. Due to the high content of structural proteins (α,β,γ-crystallin proteins) in the lens (30–40% of its wet weight), and the small size of lens tissue, it was difficult to purify and harvest a large quantity of TTase from the lens. Therefore, we resorted to use TTase gene cloning to further our research. Subsequently, the human lens *TTase* gene was cloned and purified [44], and shown to have an identical sequence to the *TTase* gene from other human tissues [45,46,47]. Recombinant human lens thioltransferase (RHLT) was produced, purified and characterized. It has a molecular weight of 11.8 kDa and displays similar structural, functional and kinetic characteristics to those of TTases from other non-ocular sources. 

Upon examining the distribution of TTase in the eye, it was not surprising to find it present in all the ocular tissues except the vitreous body. It is more concentrated in the anterior segment of the eye, including the iris, cornea epithelial layer and the lens. Such a distribution pattern coincides with the high activity of oxidative defense enzymes present in these tissues of the eye that are known to be most vulnerable to photooxidation [48].

## 6. Can Thioltransferase (TTase) Regulate Redox Homeostasis in the Lens? 

After finding the presence of TTase in the lens, we revisited the H_2_O_2_–exposed rat lens culture model of the PSSG recovery phenomenon [24] to explore the status and relationship of TTase with GSH and PSSG hoping to shed some light to this unique observation. In order to have more tissue samples for the biochemical assays. Pig lens organ culture was chosen as a model since the larger lens could be used to assay TTase activity, and contents of GSH, PSSG and PSSC in the single lens homogenate. It was observed that during the course of the first 24 h of 0.2 mM H_2_O_2_ insult, GSH loss was inversely related to PSSG elevation while TTase activity was progressively lost, closely related to the above two. However, after continuing to culture the lens in a medium without H_2_O_2_ for an additional 24 h, lens PSSG gradually decreased to the basal level, with a concomitant elevation of both GSH content and TTase activity [25], parallel to the findings of the rat lens recovery studies [24]. Similar to the rat lens studies, the pig lens lost transparency after 24 h of H_2_O_2_ exposure but regained nearly all the transparency while culture was continued in H_2_O_2_-free medium. Therefore, the findings suggest that TTase does play a role in PSSG dethiolation and likely provides redox regulation in the lens.

To further investigate the redox role of TTase in the above pig lens model, the lens was exposed in a constant level of H_2_O_2_ at 0.2 mM or 0.5 mM and examined the changes in multiple time points, within the 24 hr-exposure, in order to gain more detailed status of the TTase. With the larger lens size, it was possible to dissect the tissue into epithelial layer, outer cortex, inner cortex and the nucleus regions to compare the distribution of TTase in these areas. It was found that TTase activity was highest in the epithelial layer; a precipitous activity drop in the outer cortex (<10% of the epithelium); a further decrease in the inner cortex; and the least activity in the nucleus [49]. 

**Figure 5:**
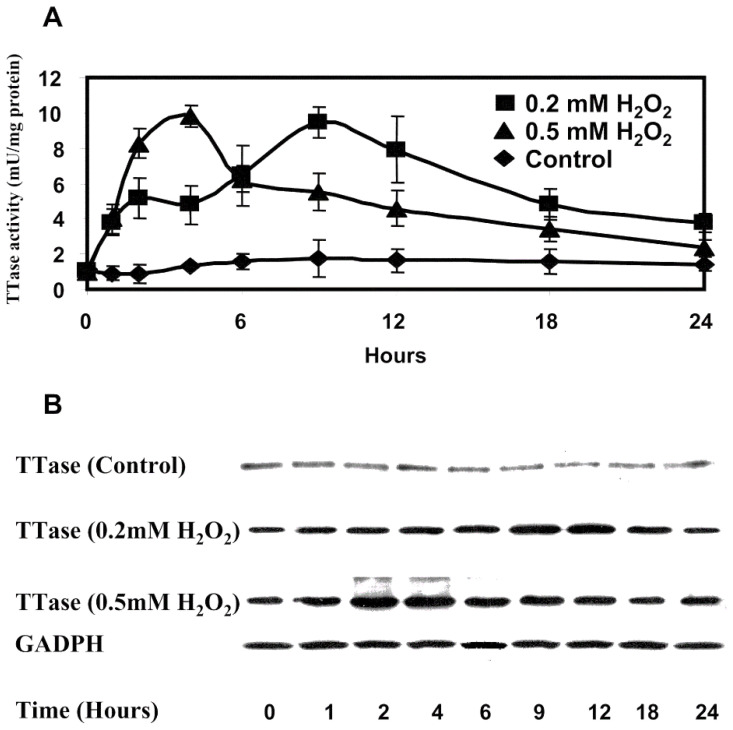
The effect of H_2_O_2_ on TTase activity and expression in cultured porcine lenses. Fresh porcine lenses were divided into three groups with three lenses per group. Group one (control) was incubated in the absence of oxidant, groups two and three were was incubated in medium containing 0.2 mM H_2_O_2_ and 0.5 mM H_2_O_2_, respectively. Lenses were taken at indicated times, epithelial layers were removed, and the total soluble fraction prepared. (**A**) The lysate of the pooled three lens epithelial layers of each group was obtained at 0, 1, 2, 4, 6, 9, 12, 18, and 24 hours and analyzed for TTase activity. Data are the average of six determinations. Error bars, SEM. (**B**) Total soluble fractions (40 μg of protein) from the lysates in (**A**) were subjected to immunoblot analysis with antibody specific to TTase. GAPDH was used as the internal control. Reprinted with permission from Moon et al., IOVS (2005); Copyright ARVO 2005 [49].

Interestingly, by doing such micro-sectional analysis, it was found that the TTase activity and its mRNA in the epithelial layer were transiently upregulated during the 24 h of H_2_O_2_ exposure. The induction was faster and stronger when a higher level of H_2_O_2_ was used (Figure 5). Such oxidative stress-induced transient upregulation also observed in thioredoxin/thioredoxin reductase in a similar manner. This redox regulating system is closely related to TTase/GSH system, which we have also found to be important redox regulator in the lens [50]. As this review will only focus on TTase/GSH system, the readers are encouraged to seek more detailed information in the 2003 review [8].

Under the above experimental condition, the spontaneous loss in GSH and lens transparency was also H_2_O_2_ concentration dependent. Such findings indicate that these two redox regulation enzyme systems of TTase/GSH and thioredoxin/thioredoxin reductase can be induced under oxidative stress, likely to protect and maintain the health of the lens in the early stage of the insult until they are, themselves, oxidized by the stress condition [49].

## 7. Cellular Functions of Thioltransferase (TTase) in Lens Epithelial Cells

The cellular function of cytosolic thioltransferase or glutaredoxin has been studied extensively in other tissues, and many review articles are available [42,51,52,53,54]. The sequence and 3-dimentional structure of thioltransferase can be found in Ogata et al. [54]. However, research on TTase in the ocular lens has been limited to the Lou laboratory. Thus, this review will only focus on the findings and advances in the lens. 

In the ocular lens, TTase is mainly present in the epithelium, thus a series of studies were carried out to examine the mechanism how TTase detoxifies oxidative stress, and how it protects other antioxidant and metabolic enzymes from oxidative damage. Cultured lens epithelial cells, either a rabbit cell line (N/N 1003A cells) or human cell line (HLE B3) were used to examine the response to oxidative stress of H_2_O_2_ in the culture medium. 

### 7.1. Properties of the Recombinant Human Lens Thioltransferase

As stated above, due to the small size of the lens organ, it was not practical to purify a large quantity of TTase from animal or human lenses. Therefore, the enzyme was cloned and purified to homogeneity [44]. The purified recombinant human lens TTase (RHLT) enzyme has a specific activity of 29.45 units/mg protein, which is nearly 30,000-fold higher than that of the TTase activity in the bovine lens [43]. It was found that RHLT requires free thiols in the active center for activity as it can be inhibited easily by iodoacetate alkylation. RHLT can be partially inactivated with H_2_O_2_ in vitro, but is quite resistant to oxidative stress and over 65–70% activity was retained even with treating up to 1.5 mM H_2_O_2_. The H_2_O_2_ inactivation can be restored by reducing agent such as GSH or DTT, as well as the enzyme system of thioredoxin/thioredoxin reductase. RHLT has a powerful dethiolating function and it preferentially dethiolates crystallin S-S-glutathione over crystallin-S-S-cysteine [34]. However, it shows the ability to reactivate the oxidation-inhibited key antioxidant enzymes such as glutathione S- transferase or glutathione peroxidase when each was inactivated by S-cysteinylation at its respective active site. RHLT can also effectively reactivate the H_2_O_2_-sensitive glyceraldehyde-3-phosphate dehydrogenase (G-3PD), a key metabolic enzyme in glycolysis [44]. The reactivation of S-thiolated GST, GPx or G-3PD could only be efficiently achieved by the catalytic function of RHLT and not by simple GSH reduction, even when physiological levels of GSH were used (1–5 mM).

TTase is not only richly present in the epithelial cells but is a highly oxidative stress resistant enzyme. Its function as a redox regulator was effectively demonstrated using both the human lens epithelial cell line (HLE B3) and the rabbit N/N 1003A cell line in culture, in which the cells were exposed to a bolus of H_2_O_2_ in serum-free media for up to 180 min. This is a very useful model as the level of H_2_O_2_ in the medium can be simultaneously monitored along with its effect to the activities of several oxidation defense enzymes. In addition, the change of GSH content and the presence of protein thiol mixed disulfides (PSSG and PSSC) can be compared. 

**Figure 6:**
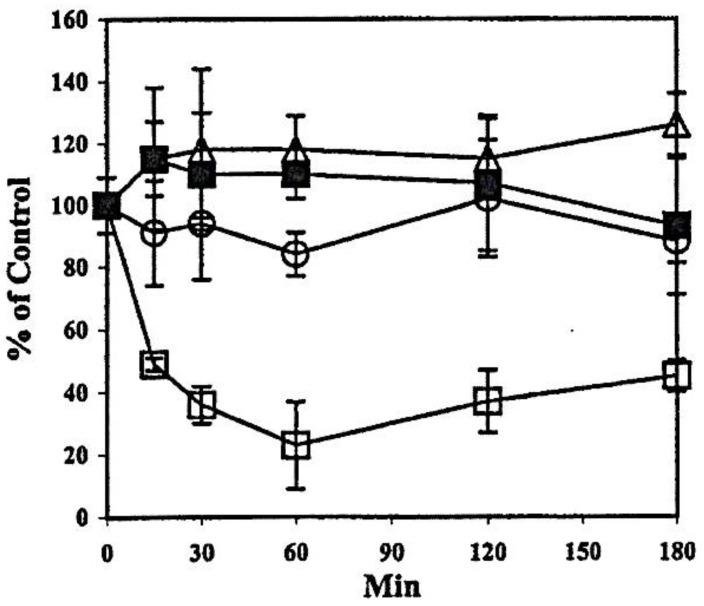
Effect of a bolus of H_2_O_2_ on the activities of oxidation defense enzymes in HLE B3 cells. The activity is expressed as % of control untreated cells in three separate experiments (mean ± S.D.). The basal enzyme activity (untreated cells) was 7.8 ± 1.3 mU/mg protein for TTase; 142.4 ± 8.0 mU/mg protein for GST; 12.8 ± 1.4 mU/mg protein for GR and 18.7 ± 4.7 mU/mg protein for GPx. -Δ- GR, ⟥ GPx, -**○**- GST, -■- TTase. Reprinted with permission from Xing and Lou, EER (2002); Copyright Elsevier 2002 [55].

During the study time course of 180 min, the HLE B3 cells could detoxify 0.1 mM H_2_O_2_ within 1 h. Cellular GSH level was quickly depleted to 30% in 30 min and recovered to 90% of its normal level at the end of 3 h. in contrast PSSG and PSSC elevated 60% and 20%, respectively, within 30 min but all recovered to near normal levels at the end of experiment [55]. Interestingly, the activities of antioxidant enzymes showed different responses (Figure 6). 

Glutathione peroxidase (GPx) was extremely sensitive losing 80% of its activity and regained to 40% by the end of 3 h. Glutathione S-transferase (GST) was less affected, lost about 20% activity but could recover completely. Glutathione reductase (GR) and TTase were not affected at all [55]. 

This unique property of TTase in oxidative stress-resistance also exhibited in a rabbit lens epithelial cell line, the N/N 1003A cells, in which TTase briefly lost less than 20% of its activity and soon completely recovered even when up to 1.0 mM H_2_O_2_ was used [56]. With such strong resistance of oxidation, it is reasonable to conclude that TTase’s dethiolation function can provide protection and can repair the oxidative stress-induced damages to other key antioxidant enzymes.

Most importantly, another interesting observation was that TTase was not only unaffected by H_2_O_2_ oxidative stress, but was upregulated and showed a transient higher activity as was found in the pig lens organ culture model [49]. Studies with human lens epithelial cells also found that oxidative stress had transiently induced both the expressions of TTase mRNA and protein level in the same cell homogenates [57]. More on this topic will be covered below under redox signaling of the lens.

### 7.2. Modulation of Lens Glycolytic Pathway by TTase

The lens is a unique tissue in the body whose energy supply depends solely on glucose supply for the glycolytic pathway, which is operated mainly under anaerobic conditions. It is known in other tissues that the enzymes operating glycolysis and the reverse pathway of gluconeogenesis are modulated by a change in the status of intracellular thiol and disulfide pools [58,59,60]. It was found that similar to other tissues, the lens cellular glyceraldehyde 3-phosphate dehydrogenase (G-3PD), a key glycolysis enzyme, is extremely sensitive to oxidative stress when H_2_O_2_ was present in the culture medium in rabbit lens epithelium N/N1003A cells, resulting in a decrease in ATP and lactate productions [61]. Additionally, G-3PD either as a pure enzyme or in the cell lysate can easily be inhibited by forming protein-SS-glutathione, and that addition of purified recombinant human lens TTase (RHLT) could dethiolate and reactivate G-3PD activity. Three other key glycolytic enzymes including hexokinase, phosphofructokinase and pyruvate kinase all showed remarkable inhibition after protein-thiol mixed disulfide formation and each could be reactivated after dethiolation by RHLT, similar to the findings in RBC [41,62]. In contrast, Fructose bisphosphatase (FBPase), a key enzyme controlling gluconeogenesis was activated after forming protein-thiol mixed disulfide, but could be inactivated after dethiolation by RHLT plus GSH. These findings [61] indicate that lens glycolysis and gluconeogenesis are both controlled by the same factors but in an opposite direction. The mechanism that glycolytic enzyme activities can be modulated by thiol-disulfide exchange according to the cellular redox status may be more important under oxidative stress for the lens to adapt to environmental change or to balance glucose metabolism to produce sufficient enzyme or reducing power. The concentrations of ATP and pyruvate in the lens cells at times may be compromised so that glucose-6-phosphate is conserved and available for NADPH production via pentose phosphate shunt, or the hexose monophosphate shunt (HMS). These findings support the earlier report of Giblin et al. [63] who found that oxidative stress induced a 2–3 fold HMS activity increase in a rabbit lens.

### 7.3. Lens TTase Can Mediate Ascorbate Recycling

TTase in many cell types has shown multifunctional properties besides catalysis of the dethiolation of protein-thiol mixed disulfides. Liver TTase displayed the ability to reduce dehydroascorbate (DHA) to ascorbic acid in vitro [64]. Such findings prompted us to examine if the lens TTase has such function. Ascorbic acid besides GSH is an important reducing agent in ocular tissues, especially in the aqueous humor. Upon oxidation, ascorbate forms DHA and H_2_O_2_. DHA is very unstable and can be irreversibly converted to products that can undergo a Maillard reaction with lens proteins and contribute to cataract formation. A series of studies [65] were carried out in lens epithelial cells, in cell-free systems, and in vivo experiments. All strongly suggest that, indeed, TTase has DHA reductase activity as demonstrated by inhibiting DHA reductase using anti-TTase antibody, and TTase overexpression in the cells increases DHA reductase activity. These findings suggest that TTase is a participant in a major ascorbate recycling system in lens epithelial cells.

## 8. Mechanism of Lens TTase Adaptive Response to Oxidative Stress and the Presence of Signal Transduction Systems in the Lens

### 8.1. How Is Thioltransferase in the Lens Being Upregulated under Oxidative Stress and What Is Its Relationship to Cell Signaling Transduction?

**Figure 7:**
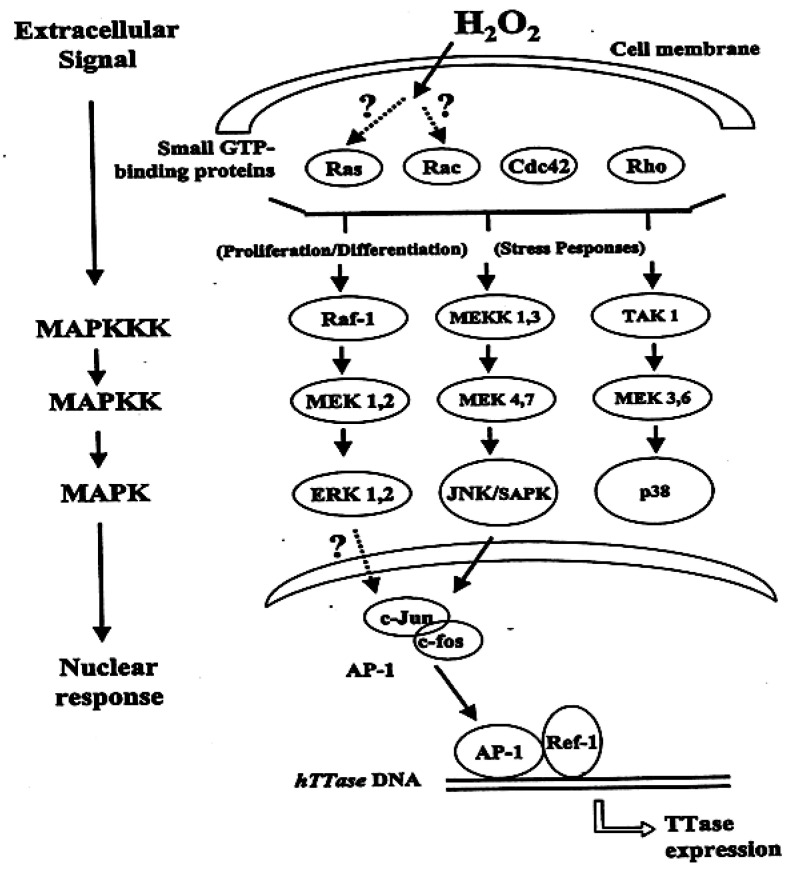
Possible mechanism for redox signaling-induced TTase expression. Ras, Rac, Cdc42, Rho are small GTP-binding proteins; MAPK: mitogen activated protein kinase; MAPKK: MARK kinase; MAPKKK: MAPKK kinase; Raf-1-MEK 1,2-ERK1,2 represents the mitogenic pathway; MEKK 1,3-MEK 4,7-JNK/SAPK represents stress/mitogenic-pathway; TAK 1-MEK 3,6-p38 represents stress-associated pathway; AP-1 represents the transcription factor consists of c-Jun and c-fos heterodimer; ref-1 represents the redox sensor for AP-1; *httase*: human thioltransferase gene and TTase: thioltransferase. Reprinted with permission from Lou, PRER (2003); Copyright Elsevier 2003 [8].

TTase could be transiently upregulated under oxidative stress as observed either in vitro organ culture with bovine lens exposed to H_2_O_2_ [49], or in vivo studies with mice exposed to ultraviolet radiation [66]. The question is “what is the mechanism that induces thioltransferase activity and its expression?” To explore such an intriguing question, human lens epithelial cell line (HLE B3) was subjected to H_2_O_2_ oxidative stress. We found that the cells exhibited a transient induction of both *hTTase* activity and mRNA gene expression [57]. Further analysis revealed that *hTTase* gene’s 5′ binding region has several putative transcription factor-binding sites, similar to the binding sites of AP-1 transcription factor, and the binding occurred in an oxidation-dependent manner [67]. By using a mobility shift assay with P^32^ labeled, double stranded oligonucleotide containing the putative AP-1 binding site and nuclear lysates from normal and H_2_O_2_-treated HLE B3 cells, we found a binding complex formed between AP-1 in the lysate and the labeled nucleotide probe. The DNA binding ability of AP-1 was increased after only 5 min of H_2_O_2_ treatment, peaked at 10 min and returned to its normal basal level after 60 min. This binding pattern corroborated the TTase activity and its mRNA level in the same cells. Furthermore, c-Fos and c-Jun, the subunits of AP-1, were both found in the binding complex when probed with supershift assay using their respective antibodies. In addition, Ref-1, a co-activator to AP-1 was also found in the same cell lysate. Thus, it is quite conclusive that AP-1 plays a major role in TTase gene induction. Furthermore, the oxidative stress-stimulated AP-1 is known to bind to its target gene via a cell process of redox signaling [68,69] that is associated with a cell signal transduction of phosphorylation-dephosphorylation process. Therefore, it was not surprising to detect the presence of a transient phosphorylated c-Jun, or activated c-Jun, (P-c-Jun) in the H_2_O_2_-exposed HLE B3 cell lysates, similar to the transient AP-1-DNA binding under the same experimental conditions. The upstream signaling component of JNK for c-Jun was also detected in its activated and phosphorylated form (P-JNK), again, similar to the pattern of P-c-Jun. The brief presence P-JNK in the cytosolic lysates indicates that it was transported into the nucleus after it was phosphorylated in the cytosol (Figure 7). 

These findings suggest that oxidative stress-induced TTase upregulation is mediated by the AP-1 transcription factor, and that its expression is processed through a phosphorylation-dephosphorylation mechanism via the JNK stress response pathway. Such adaptive responses of TTase to oxidative stress in the HLE B3 cells are similar to those of inducible antioxidants in other mammalian cells [68,69]. The association of TTase with cell signal transduction will be discussed below.

### 8.2. Presence of the Signaling Transduction Systems: The Mitogenic-Response, Stress-Response and the Survival-Response Pathways in the Lens 

Our first observation that TTase may be involved in cell signaling was the fact that when HLE B3 cells were under oxidative stress, the cells exhibited a transient induction of *hTTase* gene expression [57], and a transcription factor AP-1 appeared to control such event in an intact cell [67]. How an intact HLE cell transduces such oxidative stress signals into the nucleus for AP-1 to function is an intriguing question. Other cell types, mainly, the mammalian cells are known to have an intricate signal transduction system, the mitogen-activated protein kinase family, known as the MAPK superfamily. Therefore, a series of studies were carried out to examine if the MAPK superfamily is present in the lens, and, if so, what are their responses to stimuli such as oxidative stress or growth factor.

In the late 1980′s and early 1990′s, it had been well established in many cell types that a signal transduction system exists in the cells to convey a message from extracellular stimuli, such as a growth factor, by binding with a tyrosine kinase at the receptor on the cell membrane to activate the receptor via phosphorylation on its tyrosine moiety [70,71]. The phosphorylated receptor in turn can interact with a target protein downstream such as Ras; the activated Ras in turn can activate several of its downstream effector systems. This results in the initiation of a classical mitogen-activated protein kinase (MAPK) cascade via the sequential tyrosine/threonine or threonine/serine phosphorylation and activation of Raf, the mitogen-activated protein kinase kinase, or generally termed MEK, and the extracellular signal-regulated kinases 1 and 2 (ERK1/2). Thus, when the signal reaches ERK, the activated phospho-ERK (P-ERK) can be translocated into the nucleus where it phosphorylates several targets of nuclear factors to initiate cellular responses, be it proliferation, differentiation or cell structure reorganization, depending on the stimulus that initially binds with the cell receptor. Figure 7 depicts the stress response and proliferation pathways, and specifically with a proposed mechanism for TTase expression under oxidative stress.

Although some past reports have indicated the signaling machinery is present in the lens, the presence of mitogenic pathways (raf-MEK-ERK cascade), the stress-response pathways (p38-SAPK/JNK cascades), and the survival pathway (PI-3K-Akt) were not thoroughly explored. To achieve this aim, pig lenses were used to examine the cellular response in the epithelial cell layer of an intact lens in culture after being subjected it to either mitogenic (10 ng/mL growth factor) or osmotic stress (30 mM galactose), and the results were compared with unstimulated control lenses [72]. These studies showed that all the key members in the MAPK superfamily and the PI-3K–Akt survival pathways are present in the lens and have participated in responding to various stimuli. Interestingly, growth factors have a differential stimulatory effect on the lens. In particular, basic-fibroblast growth factor (b-FGF) is the most potent stimulator for ERK, followed by epidermal growth factor (EGF) and insulin-like growth factor (IGF-1) while platelet derived growth factor (PDGF) and vascular endothelial growth factor (VEGF) were less active. PI-3K responded to these growth factors in an opposite order. Hyperglycemic condition stimulated only p38 but not SAPK/JNK while bFGF stimulated only SAPK/JNK but not p38. Both stimuli could activate the Raf-MED-ERK and PI-3K-Akt pathways. Furthermore, we also found that the basic signaling systems in lens epithelial cells are well regulated, and with an intercommunicating system, similar to other cell types. For instance, if one cascade is suppressed under certain condition, another pathway can be activated to compensate for the otherwise weakened signaling [73]. 

## 9. Redox Signaling in the Lens for Cell Proliferation

After establishing the presence of the MAPK superfamily and the mechanism of the oxidative stress adaptive response of the TTase gene in lens epithelial cells, our research team focused on studying the redox signaling in cell proliferation of the lens below. 

### 9.1. Association of ROS with Growth Factor-Stimulated Cell Proliferation

In recent years, many research laboratories have shown that reactive oxygen species (ROS), including H_2_O_2_, are beneficial for cell proliferation in plants and animals [74,75,76,77]. In particular, several studies have found that the basis for a growth factor to induce cell proliferation is closely associated with its ability to produce ROS in situ [78,79,80,81]. One of the targets for ROS in vivo is its participation in the reversible oxidation of certain phosphatases. Together with protein tyrosine kinase, phosphatases are responsible for maintaining a normal phosphorylation-dephosphorylation homeostasis in cell signaling. At the time when the above information became available, little or no research in this regard has been reported for the lens. 

**Figure 8:**
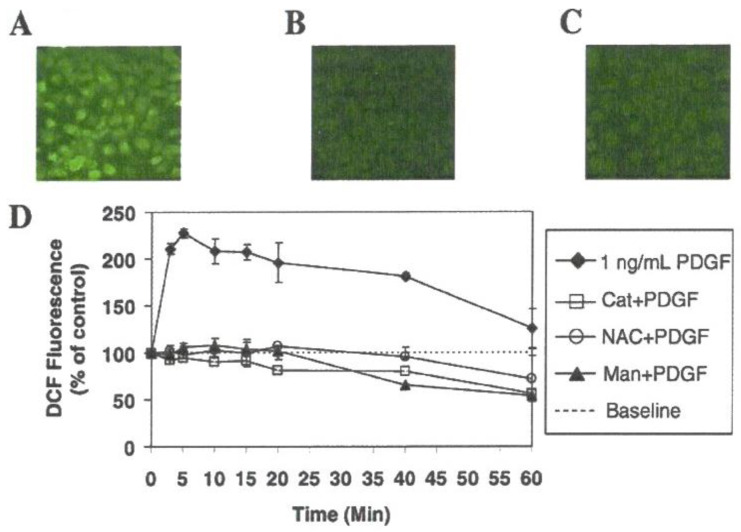
Confocal images of intracellular ROS generation upon platelet-derived growth factor (PDGF) stimulation in live HLE cells. Live HLE cells were preloaded with DCFH-DA (50 μM) to capture the ROS generated upon PDGF (1 ng/mL) stimulation. Confocal cell images represent a random field after PDGF exposure. (**A**) PDGF-stimulated cells at 10 min. (**B**) Non-PDGF-stimulated cells at 10 min. (**C**) Catalase (I mg/mL) preloaded cells at 10 min after PDGF exposure. (**D**) Inhibition of PDGF-stimulated ROS generation by antioxidants and free radical scavengers. Inhibition of the DCF fluorescence induced by PDGF (1 ng/mL) in situ is expressed as a function of time in the presence of catalase (1 mg/mL), N-acetylcysteine (NAC, 30 mM) or mannitol (Man, 100 μM). The data are expressed as the mean ± SD with *n* = 3. ♦, cells stimulated by 1 ng/mL PDGF alone with no preloading; **○**, cells preloaded with N-acetylcysteine (NAC, 30 mM); ⟥, cells preloaded with catalase (Cat, 1 mg/mL); solid triangle ▲, cells preloaded with mannitol (Man,100 mM). Reprinted with permission from Chen et al., EER (2004); Copyright Elsevier 2004 [83].

It is known that lens epithelial cells are rich in receptors for PDGF and other growth factors allowing them to continuously proliferate and differentiate into lens fibers during normal lens development and throughout the life span of a lens [82]. Therefore, PDGF-stimulated human lens epithelial cell line (HLE B3) was chosen as a model to investigate if the redox signaling system is present in the lens. The results provided the first evidence that ROS is involved in the cellular response of lens epithelial cells, and it is safe to claim that a redox-signaling pathway is present in the lens [83]. Such conclusion is based on the following findings: First, HLE B3 cells exposed to PDGF growth factor generated ROS within 10 min as visualized with a DCFH-DA fluorescence dye and confocal microscopy (Figure 8).

**Figure 9:**
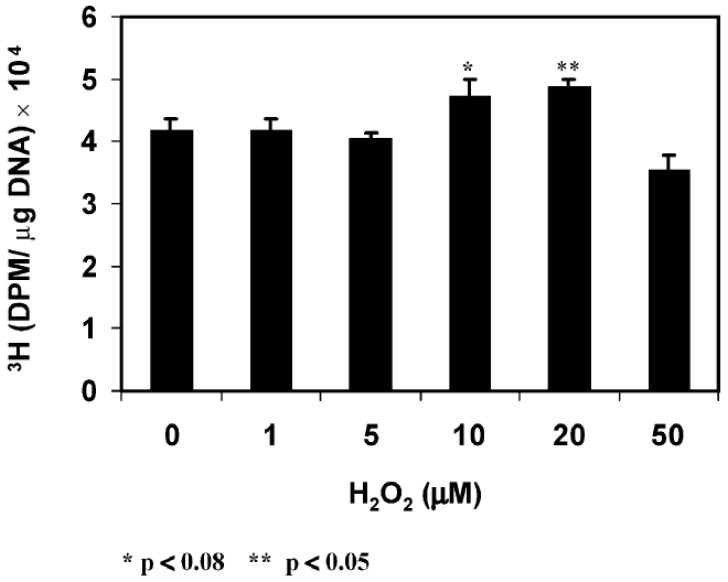
Effect of H_2_O_2_ on the proliferation of HLE B3 cells. Cells (5.25 × 10^5^) were gradually depleted from serum and used for cell proliferation by thymidine incorporation. The cells were exposed to H_2_O_2_ (0, 1, 5, 10, 20, 50 μM) in the presence of (methyl-^3^H)-thymidine (1 μCi /mL) for 1 h before harvesting for analysis. Thymidine incorporation in cells is expressed as dpm/μg DNA. The data are an average of three separate experiments (mean ± SD) with * *p* < 0.08 and ** *p* < 0.05. Reprinted with permission from Chen et al., EER (2004); Copyright Elsevier 2004 [83].

The intensity of DCFH-DA fluorescence was diminished extensively when the cells were pre-loaded with catalase or other antioxidants before PDGF stimulation. Second, PDGF-induced cell proliferation, quantified as DNA synthesis measured by (methyl-^3^H)-thymidine incorporation was inhibited by catalase. Third, PDGF activated MAP kinases (P-MEK, P-ERK1/2, P-JNK) measured by protein content with SDS-PAGE showed gradual elevation by 10 min and decreased to normal level by 20 min. Such activation was totally eliminated when catalase was present. P-p38 (survival signal) was not affected either with PDGF or PDGF plus catalase. Fourth, exogenous H_2_O_2_ at low concentration ranging 10–20 μM showed the same transient stimulatory effect as PDGF for DNA synthesis and P-ERK ½ and P-JNK productions with no effect on p38 (Figure 9). 

These studies provide two important points. First, PDGF and other growth factors can stimulate cell growth by their ability to produce ROS in situ, and second, low level of exogenous H_2_O_2_ can mimic PDGF to achieve the same cellular function. Furthermore, the extracellular H_2_O_2_ used is functional only in a very narrow range of 10–20 μM. Lower levels of 1–5 μM had no cell growth effect at all while higher range of 50 μM had damaged and suppressed cell growth. Therefore, our studies support the hypothesis of Finkel and Holbrook [84] on the importance of a balanced in situ ROS level for proper cellular function.

### 9.2. How Does a Growth Factor Induce ROS Generation In Situ for Cell Signaling in the Lens?

As stated above, growth factor, indeed, produced ROS for redox signaling in cell proliferation (Figure 8). However, the question remains where and how are the in situ ROS generated? To continue this quest our research team focused on the presence of a membrane enzyme NADPH Oxidase (NOX), which is known to generate in situ ROS for its host-defense in phagocytic cells [85]. NOX was also found in the non-phagocytic cells [86,87], including the lens [88]. The signaling that originated from growth factor or cytokine binding with the receptor at the cell surface appeared to involve several mediating points downstream before reaching the membrane NOX for ROS production. One of the key mediators for NOX activation is arachidonic acid (AA), which is released from membrane phospholipids by a cytosolic enzyme called phospholipase A_2_ (cPLA_2_). AA can respond to a variety of stimuli to accomplish its mission to stimulate NOX activity for ROS generation [86,89]. 

With the wealth of information available in the literature, it was decided to study the in situ ROS production in the lens during PDGF stimulation by examining the role of arachidonic acid and other downstream mediators using human lens epithelial cell line B3 (HLE B3) as a model [90,91,92,93]. The cellular ROS produced was observed using DCFH fluorescence dye. Our studies had confirmed the findings of Rao et al. [88], that NOX indeed is present in the lens epithelial cells. More importantly, we were able to reveal the mechanism for how NOX was activated and how ROS was produced through a series of special-designed studies. First of all, arachidonic acid (AA) was shown to play a key role by providing positive feedback during PDGF stimulation. When the cells were stimulated by PDGF or other growth factors, it was found that AA was released quickly within 5 min, and its release was dependent upon the presence of an active cPLA_2_, a specific enzyme that cleaves AA from the membrane phospholipids. Additionally, the ERK pathway appeared to control AA release, likely through regulating the bioavailability of an active cPLA_2_. Interestingly, exogenous AA itself could stimulate ROS production in the absence of PDGF. It could subsequently influence the MAPK pathways, in particular the signaling components in ERK and JNK pathways but not the p38 pathway [90]. This AA-stimulated ROS production and signal activation were similar to the results observed when PDGF was used to stimulate redox signaling in the HLE B3 cells [83]. Furthermore, PDGF-stimulated ROS production was completely eradicated by a cPLA_2_ inhibitor, indicating the AA presence is required. On the other hand, when a P-ERK ½ inhibitor was used, it could only lead to a complete elimination of PDGF-stimulated ROS generation but not AA-induced ROS formation, suggesting that ERK ½ is upstream from AA to facilitate AA release and the downstream AA-induced NOX activation. The inhibitor of NOX could completely eliminate both PDGF and AA–stimulated ROS production, thus indicating NOX is the main site for ROS production in the cells [90]. 

**Figure 10:**
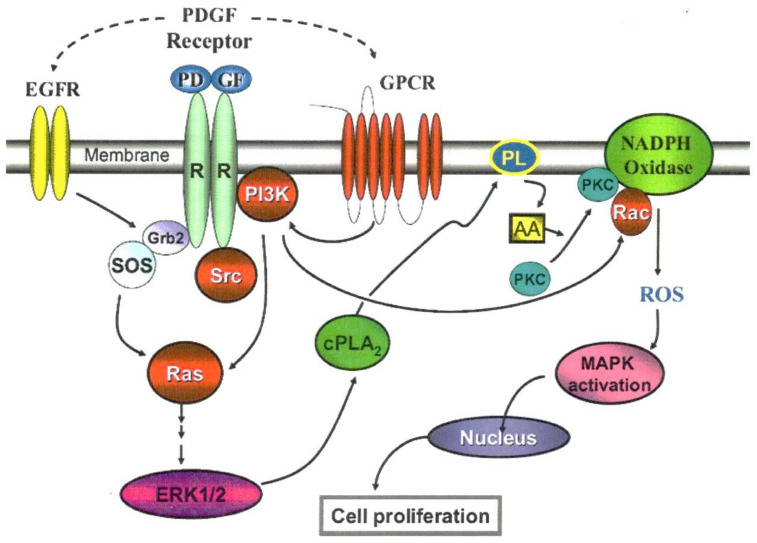
The proposed mechanism of PDGF signaling in the lens epithelial cells. The solid line indicates the known or published pathway and the dashed line represents proposed new pathway. In the figure, EGF denotes Epidermal growth factor, GPCR indicates G protein coupled receptor, PI3K indicates Phosphatidyl inositol-3-kinase, cPLA_2_ denotes Cytosolic phospholipase 2, PL indicates Phospholipid, AA denotes Arachidonic acid, PKC indicates Protein kinase C, and ROS denotes Reactive oxygen species. Reprinted with permission from Chen et al., Molecular Vision (2007); Free Access [91].

The studies described above provide a clear picture for the downstream loop during PDGF stimulation. It begins at the PDGF receptor, which signals to activate ERK ½, followed by activating cPLA_2_ to release AA from the membrane, and AA in turn can signal NOX to generate in situ ROS. The released ROS then provides MAPK activation to allow cell proliferation within the nucleus. Subsequently we have carried out a series of studies [91] and clarified that the upstream factors for PDGF mitogenic action in the human lens epithelial cells is regulated by a collective effort of several membrane-associated target proteins, similar to other cell types [94,95,96]. The stimulation begins at the PDGF receptor, followed by the concerted efforts of Src-family kinases, PI3K and the small GTP-binding proteins of Ras and Rac (Figure 10). 

### 9.3. Regulation of NADPH Oxidase (NOX) and Its Association with Cell Proliferation and Other Cellular Functions

**Figure 11:**
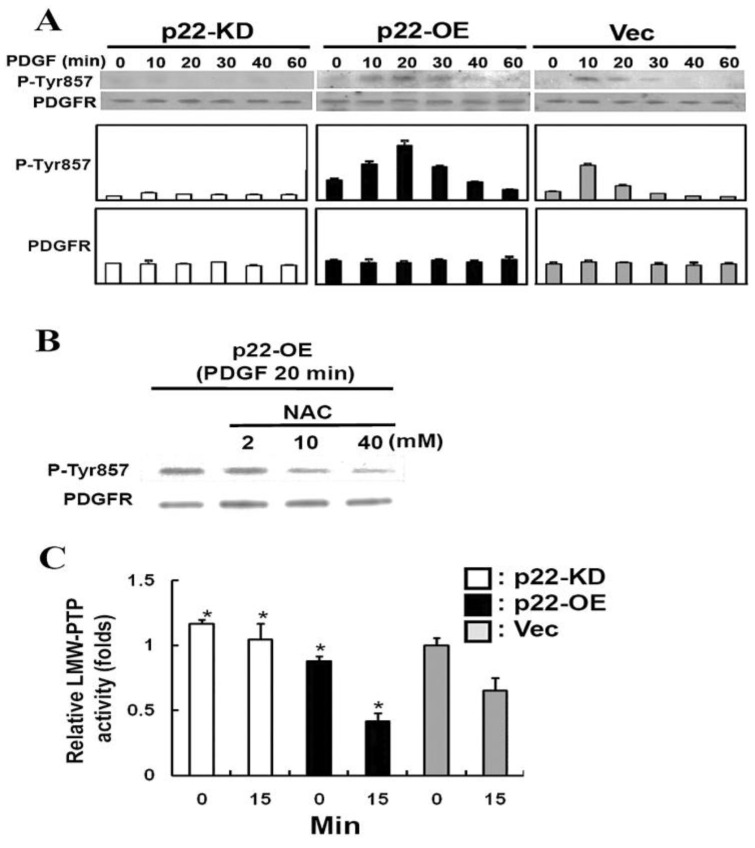
Effect of p22phox expression on the activation of PDGF receptor and LMW-PTP activity induced by PDGF in HLE B3 cells. (**A**) The Western blot analysis of PDGF-activated (phosphorylated) Tyr857 on PDGF receptor of HLE B3 cells. PDGF (20 ng/mL)-stimulated p22-KD, p22-OE, and Vec cells for 0, 10, 20, 30, 40, and 60 min were lysed and immunoblotted for P-Tyr857 PDGF receptor with anti-P-PDGFR antibody. The blot was also reprobed with anti-PDGFR antibody for control. The Western blot analysis is a typical pattern from three independent experiments. (**B**) Inhibition of PDGF-stimulated Tyr857 of PDGFR activation in p22-OE cells by antioxidant NAC. The detection of phosphorylated PDGF at Tyr857 was done by Western blot analysis in p22-OE cells. The p22-OE cells were pretreated with or without NAC for 60 min followed by the treatment of PDGF (20 ng/mL) for 20 min. Each lane contains 25 μg of cell lysate protein. The blot was reprobed with anti-PDGFR antibody as the control. (**C**) Analysis of LMW-PTP activity in PDGF-stimulated p22-KD, p22-OE, and Vec cells. The cells were stimulated with 20 ng/mL of PDGF for 0 and 15 min, and immunoprecipitated for LMW-PTP using anti-LMW-PTP antibody. Each immunoprecipitant was used for LMW-PTP activity assay. The results are the average of three independent experiments and are expressed as the mean ± SD. * *p* < 0.05 compared with Vec cells in the same treatment conditions (ANOVA). Reprinted with permission from Wang and Lou, IOVS (2009); Copyright ARVO 2009 [92].

Once our team has established that NOX is the main site for ROS production in cells stimulated by PDGF or other growth factors, an investigation was carried out to examine the role of NOX subunits as to how NOX can be activated into ROS production [92,93]. NOX has several homologues in mammalian cells, but NOX2 is the most widely distributed amongst all the isozymes. NOX2 consists of membrane-bound gp91 and p22phox, and the cytosolic components of p47phox, p67phox, Rac1/2 and p40phox [97,98]. Although Rao et al. [88] have found NOX2 and identified its subunits as p22phox and gy91phox in animal lenses, the presence of NOX2 and its function have never been explored in human lens epithelial cells. Therefore, we systematically examined the importance of the membrane subunits of p22phox and gp91phox to determine if they could regulate the activity and function of NOX. We used the strategy of manipulating the expression level of p22phox in the HLE B3 cells (Figure 11), and obtained very clear-cut answers to the mechanism of NOX activation [92]. 

**Figure 12:**
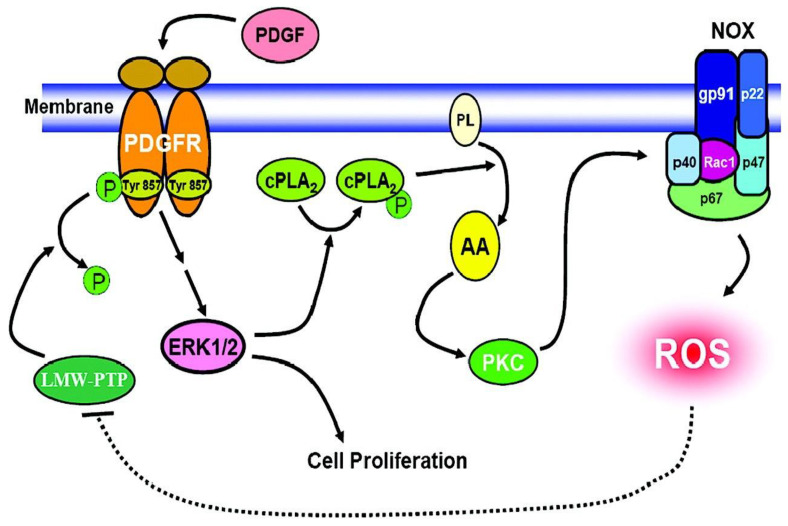
The proposed molecular mechanism of NOX regulation in PDGF-induced mitogenic signaling. Dashed line: proposed pathway; solid line: known pathway. Reprinted with permission from Wang and Lou, IOVS (2009); Copyright ARVO 2009 [92].

First of all, overexpression of the P22 gene (OE) could double NOX activity while knockdown (KD) of the gene reduced NOX activity to half in the HLE B3 cells. In accordance with the activity, OE cells generated double the amount of ROS, showed highly activated MAPK and Akt signaling cascades when the cells were stimulated by PDGF or AA, but there was almost no visible ROS or signaling effect in KD cells stimulated by either stimulant. Secondly, a p22phox-binding complex with the cytosolic subunits of p47phox, p67phox and p40phox was detected in the membrane of cells under PDGF stimulation, and the intensity of the binding complex was clearly dependent on the protein level of p22phox expressed in the cells. Thirdly, it is known that PDGF binding-induced activation of PDGF receptor requires phosphorylation of Tyr857 residue at the receptor. The phosphorylation level of Tyr857 (P-Tyr857) in PDGF-treated human lens epithelial cells was also transient, and reached plateau at 10 min, similar to the time span for the ROS released by PDGF stimulation (Figure 11). The level of P-Tyr857 was also dependent on the amount of p22phox in the cells, and could be totally eradicated when an antioxidant was present in the PDGF-treated cells. The last and most profound evidence on the importance of NOX in ROS generation was the finding that the activity of an oxidation-sensitive low molecular weight protein tyrosine phosphatase (LMW-PTP) was inversely related to the higher level of p22phox protein, such as the case in the p22phox OE cells [92]. Since LMW-PTP works opposite to protein phosphokinase (PKC), it can dephosphorylate PDGF receptor Tyr857 to prevent the PKC phosphorylation-induced mitogenic action, thus NOX produced ROS is targeting at the oxidation-sensitive LMW-PTP, allowing PDGF to proceed its mitogenic action as proposed in Figure 12. 

The topic of LMW-PTP regulation and its relationship with thioltransferase (glutaredoxin) will be described further in the following section.

## 10. Control of Redox Signaling by TTase: A Novel Physiological Function in the Lens 

Phosphorylation of tyrosine residues of intracellular proteins is one of the most important regulatory cell signaling mechanisms involved in growth factor or cytokine–stimulated cellular function. The antagonistic action of protein tyrosine kinases (PTKs, like PKC) and protein tyrosine phosphatases (PTPs, like LMW-PTP) regulates this cellular function [99]. Our laboratory has focused on studying the PTP aspect of the two regulators as we speculate PTP has a close relationship with thioltransferase [100].

### 10.1. Low Molecular Weight Protein Tyrosine Phosphatases (LMW-PTP)

Of the superfamily of PTPs, there are three isozymes known, the cdc phosphatase, dual specificity phosphatases, and the low molecular weight protein tyrosine phosphatase (LMW-PTP) [101]. For all the PTPs, under physiological conditions, the catalytic cysteine moieties have a low pKa value, and are present as thiolated anions that are highly susceptible to oxidation [102]. Therefore, they are considered as the target for in situ ROS produced during growth factor mitogenic signaling. ROS oxidation of the cysteine moiety can block the enzyme’s ability to dephosphorylate its target protein, thus a suitable level of ROS can allow the phosphorylated PDGF receptor to begin mitogenic action. Extensive studies have been carried out on the LMW-PTP in various cell types due to its involvement in cell proliferation and migration [103]. This enzyme is only 18-kDa in size. It has been found in the bovine lens and, interestingly, it has more activity in older lens epithelial cells, perhaps a reason for a slower cell proliferation or migration during aging [104]. LMW-PTP has been purified from chick lens, but the authors could never keep the enzyme in an active state due to its spontaneous inactivation by the oxygen present in the air [105].

### 10.2. Recombinant Human Lens LMW-PTP

We explored the possible relationship of TTase with LMW-PTP in the lens with the speculation that since the activation and inactivation of LMW-PTP is controlled by thiolation of cysteine moiety at the active site, then TTase may likely play some role in regulating its activity and function. Our series of studies provided several valuable new findings in the lens regarding the relationship of LMW-PTP and TTase [100]. First, we detected the enzyme as a single band using anti-human LMW-PTP polyclonal antibody from human lens epithelial cell line HLE-B3 and validated with another cell line. Then, we cloned, purified and characterized the recombinant LMW-PTP and found it to be 18 kDa in size and supersensitive to oxidation. Of the three isoforms (LMW-PTP 1, 2, and 3), LMW-PTP2 was the most sensitive to oxidation. Type 1 and type 2 isoforms were identical to those cloned from human RBC [106] and placenta [107]. Similar to the chick enzyme, the human LMW-PTP2 was spontaneously inactivated almost completely either during purification or in storage. Our mass-spectrometric data [100] clarified that the inactivated LMW-PTP2 contained one intra-molecular disulfide bond at the active site between C13–C18. Interestingly S-thiolation with either GSSG or cystine to form PSSG or PSSC in vitro could partially inactivate this enzyme, indicating disulfide bond is likely the main cause for inactivation. We also found that the inactivated enzyme could be restored in vitro easily by reductant, such as DTT.

### 10.3. Regulation of LMW-PTP by TTase in the Lens

**Figure 13:**
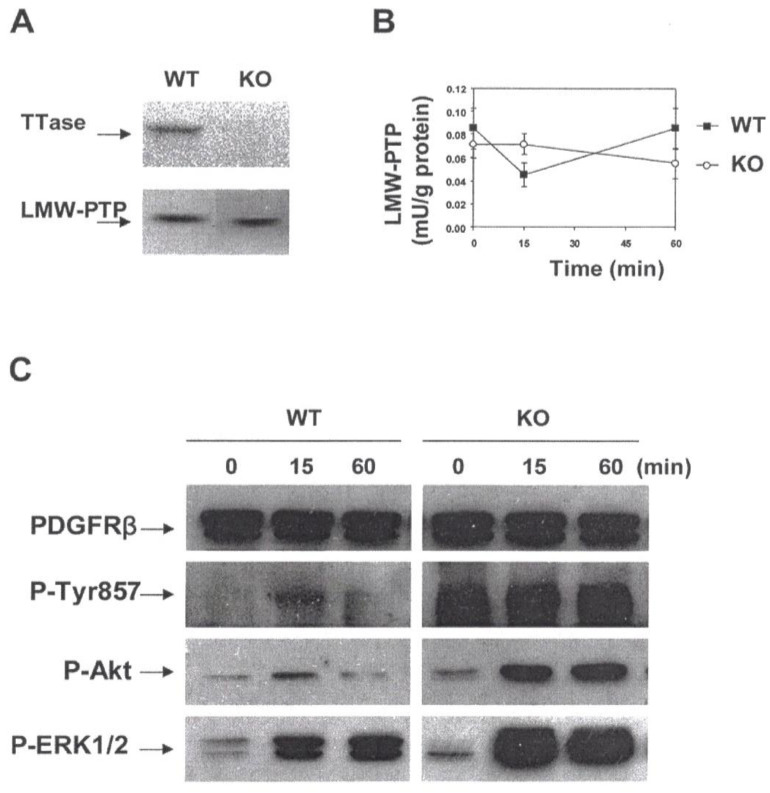
Evidence for the TTase-dependent LMW-PTP function in the mouse lens epithelial cells. (**A**). Immunoblot analysis of TTase and LMW-PTP from wild type (WT) and TTase knock-out (KO) mouse lens epithelial cells. Cell extract containing 30 g protein was resolved on 10% SDS-PAGE gel. TTase and LMW-PTP were probed with respective antibody. (**B**). PDGF-induced inactivation of LMW-PTP in WT (■) and KO (**○**) mouse lens epithelial cells. Serum-deprived cells were stimulated with PDGF (1 ng/mL) for 0, 15, and 60 min, harvested, lysed and immunoprecipitated for activity assay. Data are expressed as means ± SD, with *n* = 3. (**C**). Comparison of the PDGF-induced activation of PDGFRβ, Akt and ERK ½ in WT and KO mouse lens epithelial cells. Serum-deprived cells were stimulated with PDGF (1 ng/mL) for 0, 15 and 60 min, harvested and lysed. Cell lysate (50 g proteins) was applied on 10% SDS-PAGE gel, transferred to membrane and probed with antibodies to PDGFRβ (internal control), P-PDGFRβ (Tyr857), P-Akt and P-ERK1/2, respectively. The Western blots shown are the representative of three separate experiments. Reprinted with permission from Xing et al., BBA (2007); Copyright Elsevier 2007 [100].

The question remains that under physiological condition, what is the system that can control LMW-PTP activity, considering the fact that it plays such a critical role in redox signaling? We used primary lens epithelial cells from wild type mice and TTase knockout mice as a model and focused on studying the regulation and physiological function of LMW-PTP2, in particular the role in regulating PDGF mitogenic action. For simplicity we will refer the enzyme as LMW-PTP. Based on a series of our studies [8], TTase was proven to be an important redox regulator in the lens. Thus our approach to test if TTase plays any role in regulating LMW-PTP, and the PDGF-induced cell proliferation is a logical one. The positive results [100] in this regard can be summarized in the following. First, TTase system in vitro (TTase with GSH and glutathione reductase) restored 30–66% of the catalytic activity of LMW-PTP, depending on the amount of TTase used. GSH alone failed to do so. Second, PDGF-stimulated rapid and transient ROS production, and the activation of downstream signaling components in cultured mouse lens epithelial cells showed a reciprocal pattern of inactivation of LMW-PTP enzyme in the same cells. Due to the extreme sensitivity of LMW-PTP to oxidation, we have found that H_2_O_2_ at a level as low as 10 μM could completely inactivate the recombinant LMW-PTP enzyme. Again, this is a reasonable finding as exogenous H_2_O_2_ at such a low level could mimic PDGF cell signaling, and that the PDGF-induced intracellular H_2_O_2_ production is likely in the micromolar range of 10–20 μM [83]. Finally, the importance of TTase in LMW-PTP function was demonstrated in the primary mouse lens epithelial cells isolated from TTase knockout (KO) mice, or the TTase KO cells [100]. The key evidence was that although TTase gene deletion did not affect the expression of LMW-PTP, it did strongly attenuate the catalytic activity and its biological function during PDGF-signaling. As shown in Figure 13, TTase KO cells were unable to re-activate LMW-PTP during the 60 min of PDGF stimulation, resulting in a constantly activated or phosphorylated Tyr857 at the PDGF receptor, and the concomitantly over-activated and uncontrolled downstream targets of Akt and ERK1/2 signaling pathways [100]. 

**Figure 14:**
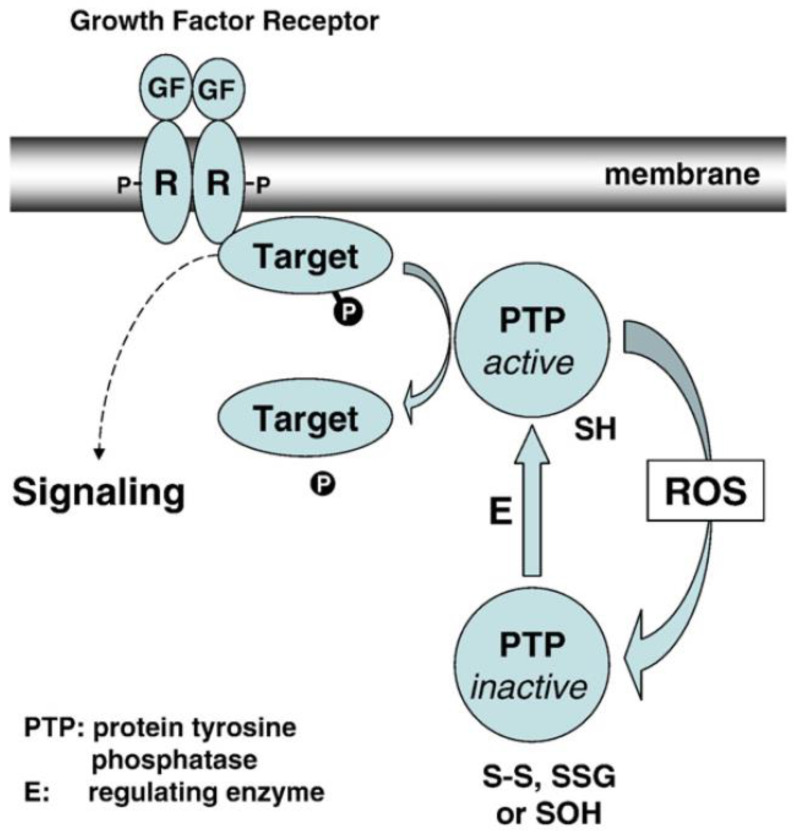
Hypothesis on the function and redox regulation of LMW-PTP in human lens epithelial cells. Growth factor (GF)-mediated mitogenic cell signaling begins by binding at the cell membrane to initiate autophosphorylation (P) at the receptor (R) and downstream target molecules (target-P) from protein tyrosine kinase. GF binding-induced in situ ROS oxidizes active PTP-SH (protein tyrosine phosphatase, such as LMW-PTP) to its inactive form (PTP with-S-S-, S-S-G or S-OH). Inactive PTP is reduced and activated back to PTP-SH by E (regulating enzyme system, such as TTase) to dephosphorylate and inactivate target proteins (target + P), allowing the completion of cell signaling. Reprinted with permission from Xing et al., BBA (2007)**;** Copyright Elsevier 2007 [100].

The function and redox regulation of LMW-PTP and its relationship with TTase in the lens epithelial cells is depicted in Figure 14. In which it is proposed that during growth factor (GF)-mediated mitogenic action in the lens epithelial cells, GF binding to the cell membrane initiates receptor autophosphorylation and ready for signaling transduction. At the meantime, the GF binding-induced ROS generation in situ (from NADPH oxidase), can oxidize and inactivate LMW-PTP to prevent dephosphorylation of the GF receptor and other target substrates. Such action would allow protein tyrosine kinase to proceed in phosphorylating and activating the downstream signaling components for cell proliferation. The unique reversible oxidant-regulated LMW-PTP in turn is then reduced and reactivated by TTase, or in combination with other reducing enzyme system, so that LMW-PTP can resume its ability to dephosphorylate and inactivate the target proteins, allowing the completion of cell signaling. 

## 11. The Importance of TTase in Cataract Prevention as Demonstrated by a TTase Gene Knockout Mouse Model

From the research we have done on lens TTase using rats as a model, we found the need to develop a TTase knockout mouse model for in depth study of the physiological function of TTase in the lens, in particular how TTase controls redox to maintain the health of a lens, and how a lack of TTase gene would affect lens transparency. We developed a homozygous TTase knockout (KO) mice in our laboratory [108] in which the KO mice developed cataract 4 months sooner than that of the wild type mice, and the lenses of KO mice showed correspondent increase in PSSG and loss in GSH levels. This model was used for the LMW-PTP research above, and the studies below. 

**Figure 15:**
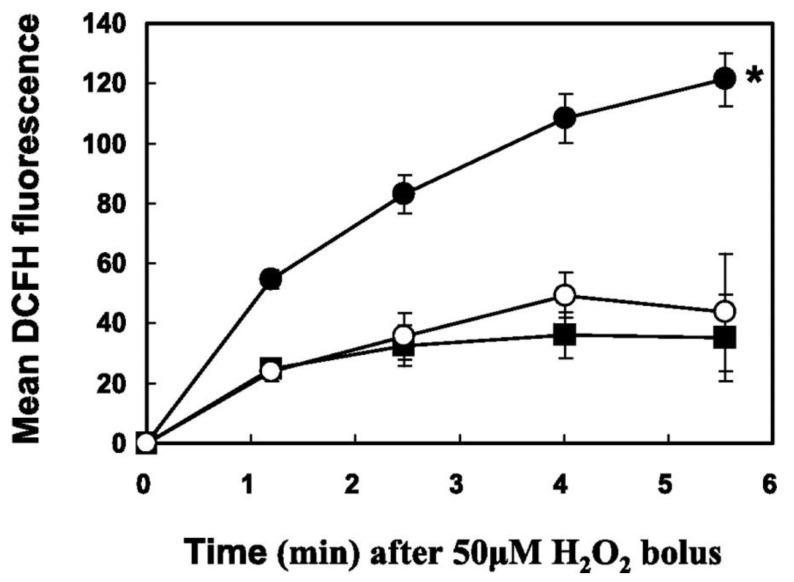
H_2_O_2_ detoxification in TTase^+/+^, TTase^−/−^, and pure TTase enzyme loaded TTase^−/−^ mouse LECs. ROS fluorescence was measured by fluorescence-activated cell sorter (FACS). The H_2_O_2_ (50 μM) treatment time course of the mean DCF fluorescence intensity was followed for *TTase*^+/+^ (■), TTase-loaded *TTase^−/−^* (O), and *TTase*^−/−^ (●). Error bars indicate SD, *n* = 5. * Significant difference from the TTase^+/+^ and TTase-loaded TTase^−/−^ cells (*p* < 0.05). Reprinted with permission from Lofgren et al., IOVS (2008); Copyright ARVO 2008 [109].

Primary lens epithelial cell cultures from both the wild type (TTase^+/+^) and the TTase knockout mice (TTase^−/−^) mice were established and compared as follows. First of all, TTase KO lens cells showed significantly lowered levels of GSH and protein thiols, with elevated content in glutathionylated proteins (PSSG). Furthermore, these cells displayed extreme sensitivity to oxidative stress. They were less viable and more apoptotic than the wild type TTase^+/+^ cells. Reloading with purified human lens TTase into the TTase^−/−^ cells could normalize the GSH and PSSG levels, as well as restore the cellular function for H_2_O_2_ detoxification [109]. These results shown in Figure 15 not only confirm the importance of TTase in thiol/disulfide regulation, but also suggest its involvement in regulating cell proliferation. 

Second, the TTase KO mouse was used for an in vivo UV radiation study, it was found that the absence of the TTase gene increased lens susceptibility to oxidative stress induced by UVR-B [110]. Finally, studies also demonstrated the protective effect of the thioltransferase gene on the in vivo UVR-300 nm-induced cataract with a protective factor (PF) at least 1.3 longer than that of the wild type, indicating that the presence of TTase gene is absolutely necessary in protecting lens clarity [111]. Therefore, the oxidative stress of either H_2_O_2_ or UV radiation confirm the importance of TTase in lens protection against cataract development.

## 12. The Status of TTase in Aging and Cataractous Human Lenses

In view of the importance of the antioxidation and physiological function of TTase in the lens, it is more relevant to examine the status of TTase in aging and cataractous lenses in humans. By doing so, we would have a possible explanation as to the relationship of TTase, the protein-thiol mixed disulfides in association with the mechanism of cataractogenesis as we have proposed nearly 20 years ago [19]. The following two studies provided strong evidence for the importance of TTase in protecting human lenses against cataract formation.

### 12.1. Effect of Age on TTase and Other Redox Regulation Systems

**Figure 16:**
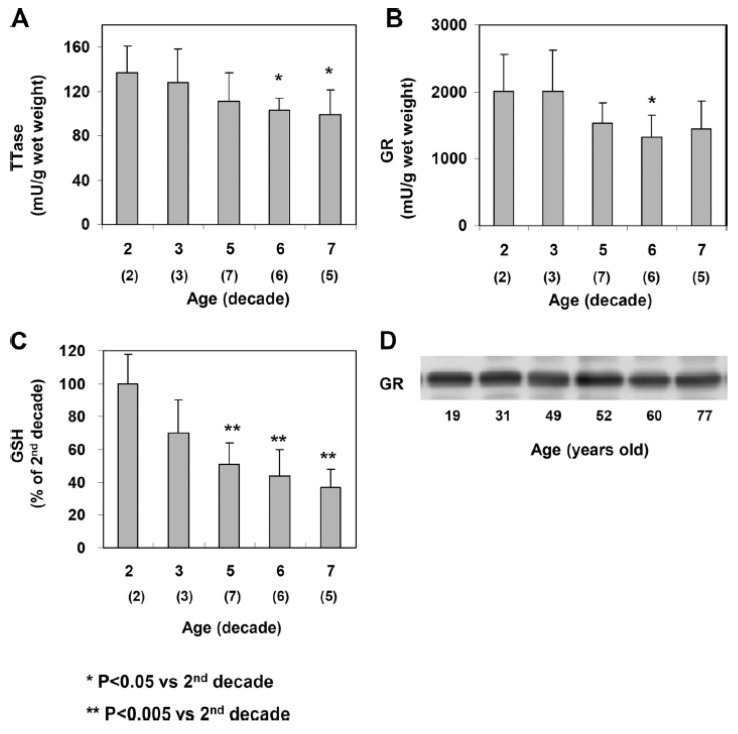
Age-dependent changes of the TTase system in human lenses. Twenty-three normal human lenses, divided into second, third, fifth, sixth, and seventh decades, were used for the study. The data are shown as mean ± SD, with the number of lenses indicated as (n) in each decade. (**A**) TTase activity in mU/g lens wet weight. (**B**) GR activity in mU/g lens wet weight. (**C**) GSH level expressed as percentage of second decade. (**D**) Homogenates from 19-, 31-, 49-, 52-, 60-, and 77-year-old lenses were selected for immunoblot analysis for GR with a specific anti-GR antibody. The blot shown is a representative of three separate analyses. Reprinted with permission from Xing and Lou, IOVS (2010); Copyright ARVO 2010 [112].

As age has been established as a major risk factor for cataract formation due to the longer period of oxidative stress exposure and the lower oxidative defense capabilities in the aging lenses. We have conducted a comprehensive, comparative study on the status of enzymes responsible for protein thiol oxidation repair, including glutaredoxin system and the thioredoxin systems in normal human lenses at ages from the second, third, fifth, sixth to seventh decades [112]. It was shown that indeed, both systems become less efficient during aging from 20 to 70 yrs. In comparison to the 20-year-old, the 70-yrs-old lenses lost nearly 30% of TTase and glutathione reductase activities, and nearly 60% of GSH content (Figure 16).

Similar losses in thioredoxin and thioredoxin reductase were observed while the key enzyme in glycolysis glyceraldehyde 3-phosphate dehydrogenase (G-3PD) suffered nearly 50% activity loss during the 50 years of aging. Although the oxidation repair system suffered modest loss in comparison to GSH level and G-3PD activity, however, the severely depleted GSH would affect the proper function of TTase, causing unreduced PSSG to accumulate in the lens that may affect the protein conformational changes with protein–protein aggregation as a severe consequence. Furthermore, the loss in G-3PD would deplete ATP production in the aging lens that can make the lens more vulnerable to external stress.

### 12.2. Effect of Lens Opacity on TTase and Other Redox Regulating Systems

**Figure 17:**
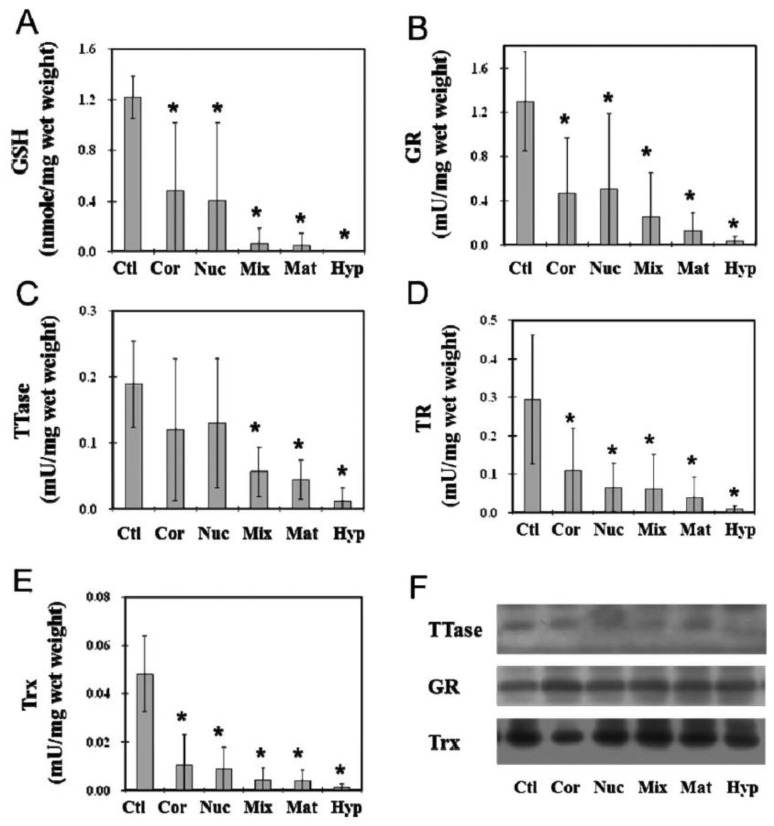
Comparison between thiol repair enzyme activities in normal nucleus and those in ECCE cataractous lenses. ECCE (extracapsular cataract extraction) procedure obtained nuclear portions of nine normal, clear human lenses (Ctl) and whole tissues of human ECCE cataractous lenses of different types of cataracts were used. The cataractous lens samples included 28 cortical (Cor), 26 nuclear (Nuc), 17 mixed cortical and nuclear (Mix), 17 mature cataracts (Mat), and 9 hypermature cataracts (Hyp). Each lens preparation was assayed for GR, TR, TTase, and TRx activities. An aliquot of deproteinized lens homogenate was used for GSH analysis. Data are expressed as means ± SD. * *p* < 0.05 by comparison with the control. Equal amounts of proteins from the above sample preparation were used for Western blot analysis for the protein content of TTase, GR, and Trx, using specific antibodies for TTase, GR, and Trx. (**A**) GSH level; (**B**) GR activity; (**C**) TTase activity; (**D**) TR activity; (**E**) Trx activity; (**F**) Western blot analysis of TTase, GR, and TRx in human cataractous lenses. Reprinted with permission from Wei et al., IOVS (2015); Copyright ARVO 2015 [113].

Another important study we have conducted was to examine the thiol repair systems in human ECCE (extracapsular cataract extraction) cataractous lens nuclei (lens tissue without the epithelial and outer cortical regions) with various degree of severity from cortical, nuclear, mixed, mature, and hypermature cataracts. Age-matched clear lens nuclei (same region as the cataractous samples) were used as the controls [113]. The status of redox regulating systems in the cataract tissues had never been explored before. This study has shown for the first time that both TTase and thioredoxin systems were extensively inactivated in proportion to the severity of the lens opacity. In fact, the most advanced categories of mature and hypermature cataracts were essentially depleted of all enzymatic activities in these redox systems. GSH, which is a well-known marker for the reduced status of a lens was almost totally depleted in the mixed, mature and hypermature cataracts (Figure 17).

Furthermore, the G-3PD activity and the level of ATP in these tissues were nearly depleted down to only 10-15% of the control normal lenses [113]. These findings confirm the potential role of oxidative stress in the cause of cataract development as we and others have hypothesized [4,5,8].

## 13. Conclusions and Future Directions

With the above findings, it can be concluded that an ocular lens has well developed oxidative defense systems that can protect lens transparency during the long exposure to oxidative stress lasting into ages of 60–70 years. The author’s hypothesis for the mechanism of senile cataractogenesis that protein-thiol mixed disulfide formation is the early event of oxidative damage that can lead to eventual protein aggregation and insolubilization is a likely scenario. The ability of the redox regulating enzyme thioltransferase (TTase) to dethiolate protein-S-S-glutathione both in vitro and in vivo models indicates that it could prevent oxidative stress-induced cataract progression. The accelerated lens opacification during UVA radiation in vivo in a mouse without the TTase gene adds further support for the important role of TTase in the lens.

The findings of redox signing in the lens and that TTase controls it during growth factor-stimulated cell proliferation is a very important landmark in lens research. It adds new knowledge regarding how important a physiological level of ROS is to cell proliferation, unlike the traditional notion that H_2_O_2_ is a damaging molecule to the cells. Therefore, ROS can be good or it can be bad, depending on the amount present in the cells, and how the cells can control and regulate a proper ROS level for its growth and survival. It is similar to the Ying-Yang teaching of the famous philosopher Confucius of China. Besides the lens, other ocular tissues, such as cornea, have shown that ROS is an essential mediator for the growth factor-stimulated corneal epithelial cell proliferation, adhesion and wound healing, and that low levels of H_2_O_2_ can mimic growth factors for the same physiological function in cornea cells [114,115]. These authors did not explore the involvement of TTase in the cornea cellular functions, but with the proximity of the two in the eye, it is likely that TTase may play a similar regulatory role in the cornea epithelial cells as well. The research findings from the lens thus may be applicable to the non-lens ocular tissues, and research in this area is worth developing.

Besides GSH and TTase covered in this review, there are other important redox regulating systems also found in the lens, namely the mitochondrial isozyme of thioltransferase, glutaredoxin2 [116], and the thioredoxin-thioredoxin reductase systems [50]. Besides showing that glutaredoxin2 can regulate the mitochondrial redox balance [117], preliminary studies in the Lou laboratory have shown that fiber cell differentiation is dependent on the presence of this enzyme in the lens [118]. Therefore, the physiological function of glutaredoxin2 has a vast territory to be uncovered. The thioredoxin/thioredoxin reductase system is known to work closely with the TTase (glutaredoxin1) system in lens redox regulation [8], plus it has a specific binding protein that controls its cellular level and function [119]. Research in both enzyme systems has been carried out in some laboratories, but the effort needs to be expanded further for more discoveries.

As for the future redox regulation research in the lens field, this author would encourage topics in the following areas for consideration. First, the role of cysteine and the protein-SS-cysteine mixed disulfide in relationship to lens transparency. For instance, why is free cysteine concentrated in the nuclear region of the lens, why is the PSSC formation tightly associated with advanced lens opacification? Second, what is the role of redox regulation in cell differentiation, cell- to -cell communication and lens development? Finally, as LMW-PTP has been shown to be more abundant in older lenses, does it control the lens aging process? If so, can any therapeutic process be adapted to suppress the activity or the expression of this enzyme to slow down lens aging and perhaps to afford better vision in the older population?
